# 

**Published:** 1817-11

**Authors:** 


### LECTURES.

Mr. GAULTER will deliver, in the ensuing season, two Courses of Lectures on the Physiology of the Human Body, at No. 10, Frith-street, Soho-square. The Lectures will be given on Monday and Thursdays, at a quarter-past eight o'clock, after the surgical lectures are concluded. The introductory lecture of the first course began on Thursday the 9th of October.

Mr. CURTIS will commence his next Course of Lectures on the Anatomy, Physiology, and Pathology of the Ear, on Thursday, November the 13th, at seven o'clock in the evening.

---

### CATALOGUE OF MEDICAL BOOKS.

Suggestions for the relief of the Sick Poor, and the improvement of the Medical Profession, in Great Britain. By John Dunn, M. R. C. S., Surgeon, Pickering, Yorkshire. 8vo. 1s.

A Practical Enquiry into the Causes of the frequent Failure of the Operations of Depression, and of the Extraction of the Cataract, as usually performed, with the Description of a Series of new and improved Operations, by the Practice of which most of those Causes of Failure may be avoided; illustrated by Tables of the comparative success of the new and old modes of practice. by Sir Wm. Adams, &c. &c. &c. 8vo. 16s.

A Letter to the Right Honourable and Honourable the Directors of Greenwich Hospital, containing an Exposure of the Measures resorted to by the Medical Officers of the London Eye Infirmary for the Purpose of retarding the Adoption and Execution of Plans for the Extermination of the Egyptian Ophthalmia from the Army, and from the Kingdom; submitted for the approval of Government, by Sir William Adams. 8vo. 3s. 6d.

A System of Chemistry, in four volumes. by Thomas Thomson, M.D. &c. &c. &c. 3l.

Aphorisms, illustrating Natural and Difficult Cases of Accouchement, Uterine Hemorrhage, and Puerperal Peritonitis. By Andrew Blake, M.D. 8vo. 5s. 6d.

A Sequel to an Essay on the Yellow Fever, principally intended to prove, by incontestable Facts and important Documents, that the Fever called Bulam, or Pestilential, has no Existence as a Distinct or Contagious Disease. By E. N. Bancroft, M.D. &c. &c. &c. 8vo. 14s.

Pharmacopœia Collegii Regii Medicorum Edinburgensis. 8vo. 10s. 6d.

A Letter to Professor Stewart, on the Objects of General Terms, and on the Axiomatical Laws of Vision. By J. Fearn, Esq. 4to. 5s.

---

### *NOTICES TO CORRESPONDENTS.*

*We have to acknowledge favours from* Dr. ROBERTSON, Mr. CRAWFORD, Mr. EDGELL, *and several others, which will appear in our next. The great length and number of our biographical articles must plead our apology for deferring critiques on several works, with the receipt of which we have been honoured, and for which we shall assign a larger portion of the succeeding Number.*

